# Zunahme teledermatologischer Konsultationen bei Verdacht auf Tinea capitis – Beobachtung fälschlich identifizierter Naevi flammei

**DOI:** 10.1111/ddg.15896_g

**Published:** 2026-02-05

**Authors:** Julian Kött, Johannes Dupont, Katharina Langen, Christian Drerup

**Affiliations:** ^1^ Klinik und Poliklinik für Dermatologie und Venerologie Universitätsklinikum Hamburg‐Eppendorf, Hamburg; ^2^ doctorderma‐Online Hautarzt, Hamburg; ^3^ Klinik für Dermatologie Venerologie und Allergologie Universitätsklinikum Schleswig‐Holstein, Campus Kiel

Sehr geehrte Herausgeber,

Wir beobachten teledermatologische Konsultationen, die bei unserem teledermatologischen Dienst „doctorderma“ von Patienten eingereicht werden, die erwähnen, dass sie über die Entwicklung einer Tinea capitis im Haar besorgt sind. In Q3/24 handelte es sich zunehmend jedoch nicht um eine Pilzinfektion, sondern um einen gutartigen Naevus flammeus (Feuermal), der typischerweise im Nacken auftritt.

Tinea capitis erfährt eine steigende Aufmerksamkeit von Dermatologen weltweit und in Deutschland seit 2023.^1^, ^2^, ^3^, ^4^ Bislang waren in Deutschland *Microsporum canis*, *Trichophyton mentagrophytes* und *Trichophyton benhamiae* die drei häufigsten Erreger, die bei Tinea capitis gemeldet wurden, aber *Trichophyton tonsurans* breitet sich weiter aus. Eine monozentrische Beobachtungsstudie der Technischen Universität München meldete einen Anstieg von *Trichophyton tonsurans* von 33,3% (zwischen 2019 und 2021) auf 68,8% im Jahr 2022.^2^ In unserer Online‐Klinik erfassen wir die Tinea capitis zwischen 0,1% (n = 1) in Q1/2023 (Q1‐Quartal 1) und Q2/2023 bis 0,58% (n = 11) aller Diagnosen (Tabelle 1).

**TABELLE 1:** Prozentualer Anteil von Tinea capitis und Naevi flammei an Kopf und Hals.

	Q 1/23	Q 2/23	Q 3/23	Q 4/23	Q 1/24	Q 2/24	Q 3/24
n (Anfragen im Zusammenhang mit Tinea capitis)	0,10% (1)	0,58% (11)	0,64% (15)	0,59% (17)	0,55% (17)	0,50% (25)	0,87% (50)
n (Tinea capitis)/ n (total)	0,10% (1)	0,58% (11)	0,60% (14)	0,52% (15)	0,52% (16)	0,46% (23)	0,66% (38)
n (Naevi flammei)/ n (total)	0 (0)	0 (0)	0,04% (1)	0,07% (2)	0,03% (1)	0,04% (2)	0,21% (12)

*Abk*.: Q, Quartal

Die Beobachtung, dass sich Patienten mit der Frage nach einer „Pilzinfektion nach Friseurbesuch“ direkt an uns wenden, korreliert deutlich mit der (sozial‐)medialen Berichterstattung zu diesem Thema. Erstmals berichteten große Tageszeitungen im Juli 2024 darüber (spiegel‐online.de: „Ausbreitung von Hautpilzinfektionen durch Trendfrisuren“, 06.07.2024; bild.de: „Gefährliche Pilzinfektion der Haut – Mediziner warnen vor Trendfrisuren“, 15.07.2024). In den sozialen Medien wurde das Thema bereits im April 2024 aufgegriffen, insbesondere durch Videos auf TikTok und Instagram, die als „viral reels“ Millionen von Aufrufen erzielten. Diese Berichte scheinen zu einem Anstieg der dermatologischen Konsultationen aus Angst vor Tinea capitis beigetragen zu haben.

In der überwiegenden Mehrheit der Fälle berichten die Patienten, dass sie einen rötlichen oder rosafarbenen Fleck auf ihrem Hinterkopf bemerken, den sie vorher noch nie gesehen haben. Meistens fällt dies mit einem kürzlichen Haarschnitt zusammen, vor allem mit einem, der den Nacken freilegt, wie etwa eine Glattrasur oder ein so genannter „Fade“. Der Naevus flammeus kann zwar schon seit der Geburt vorhanden sein, bleibt aber oft unbemerkt, bis die Haare in diesem Bereich deutlich gekürzt werden. Nach der Entdeckung der Hautveränderung sind viele Patienten beunruhigt und befürchten, dass sie sich mit Tinea capitis angesteckt haben.

In Wirklichkeit ist die Tinea capitis zwar eine bekannte Kopfhauterkrankung bei Kindern und Erwachsene,^5^ sie weist jedoch typischerweise ausgeprägtere Symptome wie Haarausfall, schuppige Stellen und Juckreiz auf, die typischerweise nicht mit einem Naevus flammeus in Verbindung gebracht werden.^6^, ^7^

Seit Q1/2023 haben wir 136 Fälle überprüft, in denen Patienten nach einem Haarschnitt Bilder ihres Nackens einreichten, da sie den Verdacht auf eine Pilzinfektion äußerten. In allen Anträgen wurde dieser Verdacht explizit angegeben. In 13,2% (18 von 136 Verdachtsfällen) haben unsere Dermatologen teledermatologisch bestätigt, dass es sich bei den Läsionen in Wirklichkeit um angeborene vaskuläre Nävus flammeus handelt, die völlig gutartig und unbedenklich sind. Der Anstieg dieser Fälle war von 0,04%–0,07% zwischen Q3/2023 und Q2/2024 auf 0,21% in Q3/2024 zu beobachten (Tabelle 1). Dies scheint eng mit der aktuellen Medienberichterstattung über Pilzinfektionen im Zusammenhang mit Friseurbesuchen zu stehen.

Die Patienten scheinen zunehmend auf Veränderungen ihrer Haut zu achten, insbesondere nach Haarschnitten. Insgesamt wirkt sich diese Sensibilisierung positiv auf die dermatologische Gesundheit aus, vor allem im Hinblick auf potenziell infektiöse Erkrankungen. Auch wenn die hier beschriebenen Fälle Fehleinschätzungen darstellten, hätte eine frühzeitige medizinische Aufklärung vermutlich dazu beitragen können, die weite Verbreitung von Tinea capitis einzudämmen. Das Aufklärungspotenzial der „traditionellen“ und sozialen Medien ist hierbei bemerkenswert. Der Anstieg der Konsultationen im Zusammenhang mit Tinea capitis verdeutlicht jedoch zugleich, dass mediengesteuerte Gesundheitssorgen bisweilen zu weit verbreiteter Verunsicherung und zur Verwechslung benigner Hauterkrankungen führen können.

Abschließend möchten wir unsere Kollegen in der Dermatologie und in den Medien ermutigen, zusammenzuarbeiten, um sicherzustellen, dass Botschaften zur öffentlichen Gesundheit verantwortungsbewusst vermittelt werden und dass die Patienten mit genauen Informationen versorgt werden. Konkret sollte neben der Gefahr einer Tinea capitis auf mögliche Differenzialdiagnosen wie Naevi flammei in den Print‐Medien und sozialen Netzwerken hingewiesen werden.

## DANKSAGUNG

Open access Veröffentlichung ermöglicht und organisiert durch Projekt DEAL.

## INTERESSENKONFLIKT

JK und KL erhielten Honorare von doctorderma‐Online Hautarzt. JD ist Mitarbeiter von doctorderma‐Online Hautarzt, CD ist Gründer und Geschpäftsführer von doctorderma‐Online Hautarzt.
